# Knowledge, Attitude and Practice Related to Vitamin D and Its Relationship with Vitamin D Status among Malay Female Office Workers

**DOI:** 10.3390/ijerph16234735

**Published:** 2019-11-27

**Authors:** Nor Aini Jamil, Nurul Nadiah Shahudin, Nur Surfina Abdul Aziz, Chew Jia Qi, Wan Az Aleesa Wan Aminuddin, Arimi Fitri Mat Ludin, Kok-Yong Chin, Zahara Abd Manaf, Norlida Mat Daud

**Affiliations:** 1 Dietetics Program, Faculty of Health Sciences, Universiti Kebangsaan Malaysia, Kuala Lumpur 50300, Malaysia; nadiah5787@gmail.com (N.N.S.);; 2 Faculty of Sports Sciences & Recreation, Universiti Teknologi MARA (UiTM) Cawangan Pahang (Kampus Jengka), Bandar Jengka, Pahang 26400, Malaysia; 3 Biomedical Science Program & Centre for Healthy Ageing & Wellness, Faculty of Health Sciences, Universiti Kebangsaan Malaysia, Kuala Lumpur 50300, Malaysia; 4 Department of Pharmacology, Faculty of Medicine, Universiti Kebangsaan Malaysia, Cheras 56000, Malaysia; 5 Centre for Biotechnology and Functional Food, Faculty of Science and Technology, Universiti Kebangsaan Malaysia, Bangi 43600, Malaysia

**Keywords:** vitamin D, KAP, sunlight exposure, office workers, female

## Abstract

This study assessed knowledge, attitude and practice (KAP) related to vitamin D and its relationship with vitamin D status among Malay female office workers. A total of 147 women aged between 20 and 55 years were recruited from a university in Kuala Lumpur. They answered questionnaires related to KAP on vitamin D, sun exposure, dietary vitamin D intake and physical activity. Serum 25-hydroxyvitamin D (25OHD) was analysed using an enzyme-linked immunoassay. Nearly half (45%) of the subjects had good knowledge but moderate attitude (76%) and practice (84%) towards sunlight exposure and dietary vitamin D intake. Median serum 25OHD was 34.1 nmol/L with the majority (91%) had vitamin D insufficiency (25OHD < 50 nmol/L). Knowledge was weakly associated with attitude (*r* = 0.29, *p* < 0.001) but no association was found between knowledge and practice (*r* = 0.08, *p* = 0.355) nor attitude and practice (*r* = −0.001, *p* = 0.994). Serum 25OHD was positively associated with sunlight exposure (*r* = 0.22, *p* = 0.008) and dietary vitamin D intake (*r* = 0.37, *p* < 0.001). It can be implied that this group is at increased risk of low bone health status, which highlights the needs of public health campaigns to improve their vitamin D status.

## 1. Introduction

Vitamin D is synthesised when the skin is exposed to UVB radiation. Another source of vitamin D is from food. However, foods that are naturally rich in vitamin D are limited and not widely consumed [1]. Geographical location of Malaysia provides ample sunlight to the country throughout the year. Yet, vitamin D insufficiency (defined as serum 25-hydroxyvitamin D (25OHD) < 50 nmol/L) has been reported in different subgroups including children, women and urban population [2,3,4,5]. Low sunlight exposure with minimal body parts exposed when outdoors (face and hands only) and limited dietary vitamin D intake have been reported in a longitudinal study in Kuala Lumpur, Malaysia [6]. Based on ethnic groups, the Malays in Malaysia had higher risk of vitamin D insufficiency due to their religion and cultural practices [2,7,8,9].

Vitamin D was found to be positively associated with knowledge and practice among post-menopausal women in a rural area in Malaysia [10]. Yet, previous studies in Vietnam [11] and Hong Kong [12] reported that a high level of awareness on vitamin D did not contribute to positive practice towards vitamin D. There are limited studies regarding the knowledge, attitude and practice related to vitamin D among pre-menopausal women in Malaysia. As osteoporosis is a chronic condition which is accelerated after menopause, early screening and awareness are important for the prevention of this disease in late adulthood. This study aimed to assess the knowledge, attitude and practice on vitamin D and its relationship with vitamin D status among female office workers in Kuala Lumpur, Malaysia.

## 2. Materials and Methods

This study employed a cross-sectional design and was conducted at a university in Kuala Lumpur, Malaysia, between March and July 2019. Malay female office workers aged 20 to 55 years were invited to participate through emails and posters. Potential participants were excluded if they had diseases known to affect vitamin D status including chronic liver disease, chronic renal disease and hypo- or hyperthyroidism. Pregnant, lactating or menopausal women were also excluded from this study. This study was conducted according to the guidelines laid down in the Declaration of Helsinki and all procedures were approved by the Research Ethics Committees of Universiti Kebangsaan Malaysia (UKM PPI/111/8/JEP-2019-116). All participants gave their written informed consent prior to participating in the study.

The demographic questionnaire covering information on age, marital status, education level, occupation and household income was self-administered by the participants. Weight and height were measured using a calibrated digital weighing scale (SECA 800, Hamburg, Germany) and stadiometer (SECA 217, Hamburg, Germany).

### 2.1. Skin Colour

Skin colour of the subjects was determined on the upper inner right forearm using a handheld chromameter (CR-400, Konica Minolta, United States). Calibration against a standard white tile was carried out before each measurement. Measurement was recorded for lightness (L*), the ‘red-green’ (a*) and the yellow-blue (b*) axes of colour based on the Commission International De L’Eclairge L*a*b* system [13]. The individual typology angle (ITA) was calculated [14] and the skin was classified into different Fitzpatrick skin types based on the ITA angles [type I (very light) > 55° > type II (light) > 41° > type III (intermediate) > 28° > type IV (tanned) > 10° > type V (brown) > −30° > type VI (dark)] [15].

### 2.2. Dietary Vitamin D Intake

Dietary vitamin D intake was assessed using a modified food frequency questionnaire (FFQ) for vitamin D intake among urban pregnant women in Malaysia [16]. The modification included the exclusion of maternal supplementation list. Participants were asked to determine the frequency and amount of intake as well as the brand name for commercial food products. Food portion sizes were determined by household measurement. Since the Malaysian food composition database does not contain data for vitamin D [17], the vitamin D content of raw foods was obtained from the United States Department of Agriculture (USDA) Standard Reference Database (Nutritionist Pro™ Software, First DataBank, Inc., San Bruno, CA, USA), while the information for commercial products was taken from the packaging labels. Total dietary vitamin D intake (g/day) was determined by multiplying vitamin D content of foods, portion size and frequency of consumption per week.

### 2.3. Sunlight Exposure

Sunlight exposure and behaviours were assessed by a questionnaire adapted from a previous study [4,18]. Participants were asked about their duration and frequency (per week) of doing outdoor activities in five domains, namely activity at work, transportation, home, exercise and others. In each domain, participants were asked to report their usual outdoor attire and use of sunscreen and umbrellas. The “Rule of Nine” was used to estimate the proportion of body surface area (BSA) exposed to sunlight based on the reported attire during outdoor activity. The sun index was calculated using the following equation:

Sun index = time (hours of sun exposure per week) × BSA exposed to sunlight

### 2.4. Physical Activity

The Malay version of Global Physical Activity Questionnaire was used to determine the participants’ physical activity level [19]. This questionnaire was used to collect information on the duration and frequency of moderate and vigorous-intensity physical activity during work, transportation and leisure time. Participants were asked to report activities that only lasted 10 min or longer. Moderate/vigorous-intensity activities are described as activities that require moderate/hard physical effort and cause small/large increases in breathing or heart rate. The metabolic equivalent (MET) of task for moderate and vigorous-intensity activities were assigned as 4 and 8, respectively [17]. Total METs were derived by multiplying value of METs with frequency (days per week) and duration (minutes) for each activity. Data were cleaned based on the WHO GPAQ analysis guidelines. Participants were further classified according to their physical activity level (low, moderate and high [20].

### 2.5. Knowledge, Attitude and Practice (KAP) Questionnaire

A set of questionnaires on vitamin D-related knowledge, attitude and practice was adapted and modified from previous studies [10,11,12,13,14,15,16,17,18,19,20,21]. Content validity (*n* = 6) and face validity (*n* = 20) were conducted prior to administration of the questionnaire. The final questionnaire consists of 12 questions on knowledge of vitamin D where participants were asked to answer ‘Yes’, ‘No’ or ‘Not Sure’. One score was given for a correct answer and ‘a zero’ for a wrong or ‘Not Sure’. The attitude part consisted of 12 statements and was rated on a 5 Likert scale with response from ‘strongly disagree’ to ‘strongly agree’. The score was given from 1 to 5 with higher score for a positive attitude. The final part of practice contained 10 statements rated on a 5 Likert scale with responses of never/rarely/sometimes/often/always and scored as 1 to 5 for a good practice. The total raw scores for knowledge (ranged from 0 to 12), attitude (12 to 60) and practice (10 to 50) were converted into percentages. A score of 75% and above was classified as having good knowledge/attitude/practice; 51–74% as moderate and 50% or less as poor, respectively.

### 2.6. Blood Collection and Determination of Serum 25OHD Level

A total of 5 mL non-fasting blood sample was collected by trained phlebotomists. Blood samples were centrifuged at 3000 rpm in a refrigerated centrifuge (4 °C) for 15 min, within an hour after sampling. The serum then was pipetted into sterile Eppendorf tubes, labelled and stored at −80 °C. Serum 25 OHD concentrations were measured using immunoassay (IDS EIA, UK). Participants were further classified as having insufficient (<50 nmol/L) or sufficient (≥50 nmol/L) vitamin D, respectively [22].

### 2.7. Statistical Analysis

All analyses were carried out using SPSS Version 25.0 (IBM, NY, NY, US). Data were checked for normality using Kolmogorov Smirnov test, histogram and scatterplot. Variables were described using mean and standard deviation or median and inter-quartile range as appropriate. Categorical variables were reported with number and percentage. Spearman correlations were used to assess the association between knowledge, attitude and practice with vitamin D status as well as other lifestyle factors related to vitamin D. A *p*-value < 0.05 was decided to indicate a statistically significant.

## 3. Results

A total of 147 participants took part in this study. The majority of the participants aged between 30 and 39 years (59%) and were married (63%) (Table 1). Nearly half of the participants (45%) had diploma qualification and middle household income (47%). Participants were mostly overweight (29%) and obese (22%) with a median BMI of 26.5 kg/m^2^. Majority (61%) of the participants had skin type II (light skin colour) with no participants had skin type V (brown) and VI (dark). Most of the participants had low (33%) to moderate (37%) physical activity level.

**Figure 1:**
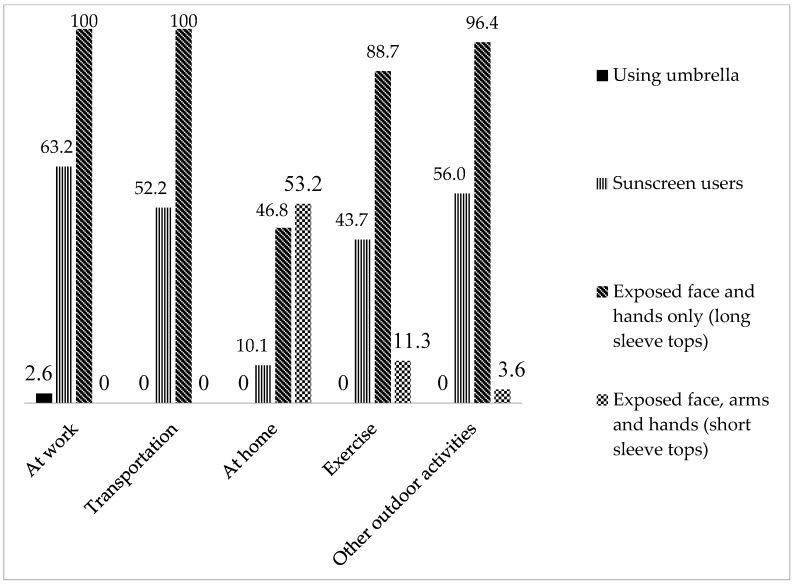
Proportion (%) using sun protection measures during outdoor activities among office female workers (*n* 147).

Majority of the participants (91%) had insufficient vitamin D status with median 25OHD concentration of 34.1 nmol/L (Table 1). Median dietary vitamin D intake (5.2 µg/d or 208 IU/d) was lower than the recommendations (15 µg/d or 600 IU/d) with only nine participants (6%) achieving the recommendations [23]. Figure 1 shows sun-protective behaviours among the participants. Most of the subjects exposed their face and hands with long-sleeve tops when outdoors except during activities at home, when they tended to wear short-sleeves. A similar trend was seen among sunscreens users, whereby there was a lower proportion of participants reported using sunscreens when doing outdoor activities at home compared with other activities.

### Knowledge, Attitude and Practice (KAP) Related to Vitamin D

Nearly half of the participants (45%) had good knowledge but the majority had moderate attitude (76%) and practice (84%) towards sunlight exposure and dietary vitamin D intake (Table 2).

The lowest scores of knowledge (either answered wrongly or ‘not sure’) were for the questions ‘vegetables are one of the food sources of vitamin D’ (71%); ‘indoor workers have low risk of vitamin D deficiency’ (61%) and ‘people with darker skin require longer sunlight exposure to synthesise the same amount of vitamin D as compared to fair skin’ (77%) (Table 3).

For responses on attitudes, 61% of the participants believed that ‘it is a must to put on sunscreen despite only exposed to the sun for a short while’ (Table 4). Another 59% of the participants were unsure if ‘the price of vitamin D fortified milk is expensive’ and whether ‘vitamin D supplementation is needed when the sunlight exposure is low’ (42%), respectively.

In terms of practice, concern about the hot weather in Malaysia was the most common reason given for sun avoidance (71%) (Table 5). Using an umbrella (4%) and taking vitamin D supplements (2%) were not popular practices among the participants.

Knowledge was weakly associated with attitude (*r* = 0.29, *p* < 0.001) but no association was found between knowledge and practice (*r* = 0.08, *p* = 0.355) nor attitude and practice (*r* = −0.001, *p* = 0.994) (Table 6). For factors related to lifestyles, the score of knowledge was negatively associated with sunlight exposure (*r* = −0.20, *p* = 0.014). Vitamin D status was weakly associated with sunlight exposure (*r* = 0.22, p = 0.008) and moderately associated with dietary vitamin D intake (*r* = 0.37, *p* < 0.001). Skin type, physical activity and anthropometric data were not associated with vitamin D status.

## 4. Discussion

This study provides insight into the current knowledge, attitude and practice towards vitamin D among Malay female office workers in an urban area in Malaysia. Although the average knowledge score (67%) in this study was higher compared to previous study among post-menopausal women in Malaysia (45 to 47% [11], their scores for attitude and practice were similar (current study vs. past study for attitude score = 70% vs. 66 to 69%; practice score = 56% vs. 59 to 62%). The difference in knowledge score could possibly be contributed by the higher representation of women with higher education attainment (diploma and higher) compared to a previous study in a rural setting [10]. In a study among medical university students in Nanjing, China, the rate of correct responses for knowledge of vitamin D was below 50% [24].

Notably, a higher knowledge level was not associated with good practice on vitamin D. None of our study participants had good practice (>75% practice score) towards vitamin D. This is reflected in their vitamin D status when a considerable high proportion of the participants (91%) had insufficient vitamin D status. There was a trend towards a higher 25OHD concentration in participants with a higher practice score; yet, this was not statistically significant (*p* = 0.08). The median 25OHD concentration in the current study (34.1 nmol/L) was also lower than previously reported among Malay women in Kuala Lumpur, Malaysia (44 to 48 nmol/L) [2,8]. Such observations could possibly due to our participants were solely amongst office workers compared to previous studies. Currently, there is no international consensus on the optimal value of 25OHD. The Institute of Medicine (2011) proposed a serum 25OHD concentration of 50 nmol/L which meets the requirement of 97.5% of the US population, even under the conditions of minimal sun exposure [22]. A higher cut-off value of ≥ 75 nmol/L was recommended by the Endocrine Society as sufficient [25]. In the UK, serum 25OHD below 25 nmol/L was used as a threshold for vitamin D deficiency [26].

Only sunlight exposure and dietary vitamin D intake were related to vitamin D status. Yet, the associations were weak to moderate. This could possibly due to the recall bias (over or underestimate) of sun exposure and dietary intake among the study participants as they were subjectively collected through questionnaires. The majority (71%) of our study participants agreed that the hot weather in Malaysia causes them to be more comfortable doing activities indoors. Such practice with limited BSA exposed when outdoors (long sleeves clothes) may contribute to the low levels of vitamin D. Furthermore, the sun index score in this study population (0.57) was lower than previously reported among urban (0.72) and rural (0.89) women in Malaysia [4] indicating a further limited sun exposure practice in this study population. An in-depth interview or a focus group discussion may help to further elucidate the reasons these female office workers continue to avoid sunlight despite knowing of its importance on vitamin D. In a qualitative study among adults in Saudi Arabia, several barriers for sun exposure observed were hot weather, living in high-rise buildings, limited public areas for outdoor activities, physical inactivities and some religious concerns such as wearing the hijab [27].

Public health bodies in Malaysia should increase awareness about the risk of vitamin D deficiency in this sunny country with extreme sun avoidance. Whilst it is difficult to accurately recommends the amount of sunlight exposure due to many factors that influence the cutaneous synthesis of vitamin D (e.g., UVB intensity, amount of time spent outdoors, skin pigmentation and skin coverage), most available recommendations so far are from the western countries [28,29], which are different in latitude, climates and population behaviours. Moreover, the recommendations suggest exposing at least 15% of BSA (at least face, hands and arms, or equivalent) which contradicted the habitual Malay females clothing style in Malaysia (10% BSA, face and hands). More local studies on sunlight exposure are warranted to accommodate such population behaviours and culture which require covering up of skin in order to make up the shortfall in vitamin D intake.

Taking dietary vitamin D supplements and oily fish were less popular among this study population, which has also been reported previously [6]. A strong relationship between vitamin D status and the consumption of fatty fish was observed among Japanese populations [30] and Asian immigrants living in Norway [31]. In Malaysia, foods fortification with vitamin D is voluntary and limited to certain dairy products and breakfast cereals. Yet, dairy products consumption among Malaysians was generally low [32].

This study was the first to investigate the KAP related to vitamin D among office female workers in Malaysia. While skin colour was assessed objectively, the possible limitation of this study is the measurement of sunlight exposure by questionnaire, which might introduce recall bias. Furthermore, vitamin D intake could have been underestimated due to the lack of vitamin D database of local Malaysian foods. This study was also limited by a small sample size, single ethnicity and the convenient sampling of female office workers. Thus, they might not be representative of the general population of Malaysian women.

## 5. Conclusions

Malay female office workers in Kuala Lumpur were generally having good knowledge but moderate attitude and practice regarding vitamin D. Vitamin D status was related to sunlight exposure and dietary vitamin D intake. Future studies should focus on strategies to improve their vitamin D status.

## Figures and Tables

**Table 1:** Sociodemographic and physical characteristics of participants (*n* = 147).

Characteristics	*n*	%	Mean	SD
**Age (years)**			36.0	6.4
18–29 years	24	16.3		
30–39 years	87	59.2		
40–49 years	31	21.1		
50–59 years	5	3.4		
**Marital status**				
Single	46	31.3		
Married	92	62.6		
Widow/Divorce	9	6.1		
**Education level**				
Up to secondary school	27	18.4		
Diploma	66	44.9		
Bachelor or higher	54	36.7		
**Household income (MYR)**				
Low (<3860)	56	38.1		
Middle (3860–8319)	69	46.9		
High (>8319)	22	15.0		
**Skin type**				
Type I (very light)	7	4.8		
Type II (light)	90	61.2		
Type III (intermediate)	42	28.6		
Type IV (tanned)	8	5.4		
**Weight (kg)**			64.8	17.4
**Height (cm)**			156.2	5.6
**BMI (kg/m^2^)**			26.5	6.6
BMI classification				
Underweight	7	4.8		
Normal	65	44.2		
Overweight	43	29.3		
Obese	32	21.8		
**Total METs (minutes/week) ^†^**			1500	2680
**Physical activity level**				
Low	46	32.6		
Moderate	52	36.9		
High	43	30.5		
**25OH(D) (nmol/L) ^†^**			34.1	15.6
**Insufficient vitamin D (25OHD <50 nmol/L)**	133	90.5		
**Total dietary vitamin D intake (ug/day) ^†^**			5.2	6.4
**No. of participants achieved recommended vitamin D intake**	9	6.1		
**Sun index score ^†^**			0.57	0.51

MYR: Malaysia Ringgit; BMI: body mass index; MET: metabolic equivalent ^†^ Median, IQR.

**Table 2:** Knowledge, attitude and practice scores and classifications among office female workers (*n =* 147).

Variable	Mean	SD	Min	Max
Knowledge score (%)	66.7	33.0	8.0	92.0
Attitude score (%)	70.0	6.1	55.0	87.0
Practice score (%)	56.2	6.3	40.0	72.0
**Knowledge classification**	***n***	**%**		
Good	66	44.9		
Moderate	43	29.3		
Poor	38	25.9		
**Attitude classification**	***n***	**%**		
Good	35	23.8		
Moderate	112	76.2		
Poor	0	0		
**Practice classification**	***n***	**%**		
Good	0	0		
Moderate	123	83.7		
Poor	24	16.3		

**Table 3:** Responses to the knowledge statements related to vitamin D among office female workers (*n* = 147).

Knowledge Statements	Correct *n* (%)	Wrong *n* (%)	Not Sure *n* (%)
1. Bone diseases such as osteoporosis are associated with vitamin D deficiency.	113 (76.9)	6 (4.1)	28 (19.0)
2. Vitamin D can be produced from sunlight.	135 (91.8)	4 (2.7)	8 (5.4)
3. In Malaysia, the ideal time for body to expose to sunlight is before 10 am.	141 (95.9)	4 (2.7)	2 (1.4)
4. The only body part that needs to be exposed to sunlight is the face.	9 (6.1)	115 (78.2)	23 (15.6)
5. People with darker skin need longer periods of time to produce vitamin D from sunlight as compared to people with fairer skin.	34 (23.1)	47 (32.0)	66 (44.9)
6. People who work in the buildings have low risk of vitamin D deficiency.	61 (41.5)	57 (38.8)	29 (19.7)
7. The use of adequate sunscreens can prevent sunlight from penetrating the skin.	99 (67.3)	29 (19.7)	19 (12.9)
8. Fish with high fat content such as salmon, tuna and sardines are among the food sources containing vitamin D.	107 (72.8)	6 (4.1)	34 (23.1)
9. Egg yolk is one of the food sources that contains vitamin D.	92 (62.6)	7 (4.8)	48 (32.7)
10. Vegetables are among the food sources containing vitamin D.	74 (50.3)	42 (28.6)	31 (21.1)
11. Dairy products are among the examples of vitamin D enriched drinks.	110 (74.8)	3 (2.0)	34 (23.1)
12. Cod liver oil is one of the examples of supplements containing vitamin D.	113 (76.9)	3 (2.0)	31 (21.1)

**Table 4:** Responses to the attitude statements related to vitamin D among office female workers (*n* = 147).

Attitude Statements	Strongly Agree/Agree *n* (%)	Not Sure *n* (%)	Strongly Disagree/Disagree *n* (%)
1. Osteoporosis is a serious bone disease.	133 (90.4)	13 (8.8)	1 (0.7)
2. I still have time to improve my bone health status.	121 (82.3)	24 (16.3)	2 (1.4)
3. Exposure to sunlight can reduce the risk of osteoporosis.	72 (48.9)	67 (45.6)	8 (5.4)
4. We need to expose our body to sunlight every day.	106 (72.1)	27 (18.4)	14 (9.5)
5. Urbanization lowers the chance to expose to sunlight.	75 (51.0)	56 (38.1)	16 (10.9)
6. Lack of public recreational park lowers the chance to expose to sunlight.	86 (58.5)	24 (16.3)	37 (25.2)
7. Lack of campaigns focusing on the benefits of sunlight prevents the production of vitamin D as needed.	123 (83.7)	14 (9.5)	10 (6.8)
8. Working full time in the building prevents the production of vitamin D as needed.	107 (72.8)	21 (14.3)	19 (12.9)
9. We do not need to do outside household chores if we have done it inside of the house.	10 (6.8)	30 (20.4)	107 (72.8)
10. The use of sunscreen is necessary every time before going out, even only for a short period of time.	90 (61.2)	34 (23.1)	23 (15.6)
11. Vitamin D supplementation is only necessary if sunlight exposure is low.	47 (32.0)	64 (43.5)	36 (24.5)
12. The price of vitamin D enriched milk is expensive.	36 (24.4)	87 (59.2)	24 (16.4)

**Table 5:** Responses to the practice statements related to vitamin D among office female workers (*n* = 147).

Practice statements	Frequently/Always *n* (%)	Sometimes *n* (%)	Rarely/Never *n* (%)
1. I always do activities outside of the house/building.	38 (25.9)	65 (44.2)	44 (29.9)
2. During the day, I walk outside of the building (not roofed).	52 (35.4)	63 (42.9)	32 (21.8)
3. I use a hat/umbrella every time when under the sun.	6 (4.1)	32 (21.8)	109 (74.2)
4. I use sunscreen on the face and hands if staying outdoors for longer period of time (more than 1 h).	56 (38.1)	33 (22.4)	58 (39.4)
5. The hot weather in Malaysia causes me to be more comfortable doing activities indoors.	105 (71.4)	37 (25.2)	5 (3.4)
6. During the day, I expose to sunlight indirectly through window.	62 (42.1)	49 (33.3)	36 (24.5)
7. I take oily fish (e.g., salmon, tuna, sardines) at least 2 times per week.	27 (18.3)	55 (37.4)	65 (44.2)
8. I eat only egg white.	32 (22.5)	34 (23.1)	80 (54.5)
9. I read nutrition information (food labels) and choose foods/drinks enriched with vitamin D.	23 (15.7)	46 (31.3)	78 (53.0)
10. I take vitamin D supplements.	3 (2.1)	11 (7.5)	133 (90.5)

**Table 6:** Associations between serum 25OHD with knowledge, attitude and practice scores and lifestyle factors among office female workers (*n* = 147).

Variable	Knowledge		Attitude		Practice		25OHD	
	*r*-Value	*p*-Value	*r*-Value	*p*-Value	*r*-Value	*p*-Value	*r*-Value	*p*-Value
Knowledge score			0.293	<0.001 **	0.08	0.355	−0.01	0.942
Attitude score	0.293	<0.001 **			−0.001	0.994	−0.04	0.670
Practice score	0.08	0.355	−0.001	0.994			0.15	0.08
25OHD	−0.01	0.942	−0.04	0.670	0.145	0.08		
Sunlight exposure	−0.202	0.014 *	−0.03	0.711	0.090	0.279	0.22	0.008 *
Dietary vitamin D intake	−0.123	0.139	0.101	0.224	0.078	0.347	0.37	<0.001 **

* *p* < 0.05; ** *p* < 0.001.
